# Microbial Community Dynamics and Natural Fermentation Profiles of Ensiled Alpine Grass *Elymus nutans* Prepared From Different Regions of the Qinghai-Tibetan Plateau

**DOI:** 10.3389/fmicb.2020.00855

**Published:** 2020-05-12

**Authors:** Zitong Ding, Jie Bai, Dongmei Xu, Fuhou Li, Yixin Zhang, Xusheng Guo

**Affiliations:** ^1^State Key Laboratory of Grassland Agro-ecosystems, School of Life Sciences, Lanzhou University, Lanzhou, China; ^2^Probiotics and Biological Feed Research Centre, Lanzhou University, Lanzhou, China; ^3^State Key Laboratory of Grassland Agro-ecosystems, College of Pastoral Agriculture Science and Technology, Lanzhou University, Lanzhou, China

**Keywords:** bacterial community, grass silage, silage microbiome, altitude, lactic acid bacteria

## Abstract

Feed deficiency during the long cold period of a year is one of the major problems that the traditional year-round animal grazing system has faced on the Qinghai-Tibetan plateau (QTP) since ancient time. Therefore, ensiling the grasses from grassland could be a desirable feeding regime to preserve high quality forage and to alleviate the seasonal unbalanced feed supply problem on this plateau. The present study was designed to investigate dynamics of bacterial community and natural fermentation quality of ensiled *Elymus nutans* collected from grasslands in four different areas with different elevations [Tianzhu County (TZ), 2965 m; Golog Prefecture (GL), 3763 m; Damxung County (DX), 4228 m, and Nagqu Prefecture (NQ), 4752 m] on the QTP. The bacterial community was characterized by using the PacBio single molecule with real-time sequencing technology (SMRT). The harvested fresh *E. nutans* grasses were ensiled in vacuum-sealed polyethylene bags for 14, 30, 60, and 90 days. Obvious differences in the epiphytic bacterial community of the fresh *E. nutans* samples from the four areas were observed, which resulted in various bacterial community dynamics and fermentation qualities of ensiled *E. nutans*. Higher fermentation quality was observed in silage samples from Nagqu than in those from the other areas (*P* < 0.05). Lactic acid bacteria (LAB) involved in fermentation of *E. nutans* from low altitude areas consisted of *Pediococcus pentosaceus*, *Lactobacillus* sp., *Leuconostoc mesenteroides*, and Lactobacillus *coryniformis*, whereas major LAB species involved in the fermentation of *E. nutans* silage from high altitudes included *L. mesenteroides*, *Lactobacillus brevis*, and *Lactobacillus* sp. Correlation analysis between bacterial composition and fermentation quality of *E. nutans* silages made from the four different areas in the QTP indicated that the LAB species responsible for silage fermentation in different areas were totally different, which was mainly due to the different epiphytic bacterial compositions in fresh *E. nutans* before ensiling. The present results provide important information on revealing the bacterial communities and fermentation quality of ensiled *E. nutans*, and on future screening of LAB isolates for making high quality silage in order to alleviate feed shortage of the traditional year-round grazing system on the QTP.

## Introduction

The Qinghai-Tibetan plateau (QTP), known as the Earth’s third pole, is the highest large plateau on the globe with a mean altitude of above 4000 m.a.s.l. and an area of 2.5 million km^2^; it has the world’s highest, largest (1.29 × 10^8^ ha) and the only year-round grazing alpine grassland (Zhou et al., 2018). Because of its high altitude and harsh environment (low temperature with annul temperature −5 to −1°C, strong ultraviolet radiation, short growing season of 90–120 days, hypoxia, etc.), agricultural cultivation is excluded in most areas of the plateau, and the main land use on this plateau has been grazing animals since the ancient times. More than 14 million yaks (*Bos gruniens*) and over 50 million Tibetan sheep (*Oviesaries*) are inhabited on this plateau, and all these domestic animals graze on the natural alpine grassland for year-round; however, feed deficiency during the long cold period of a year (November to June) is one of the major problems that the traditional animal grazing system on the plateau has faced for a long time (Zhou et al., 2017, 2018). Due to the extremely harsh environment on the plateau, forage yields in the alpine grassland are normally quite low (Thomas et al., 2016). Additionally, the increased number of domestic animals and overgrazing in the past decades even aggravated the feed shortage (Xu et al., 2019). Therefore, efficient forage preservation is considered as an important farming strategy to alleviate feed shortage of the traditional year-round grazing system on the QTP.

Ensilage is a well-accepted and efficient way for both high quality forage preservation and grassland management. During ensiling, green fodder is fermented by epiphytic lactic acid bacteria (LAB) under anaerobic condition, and in this process, most nutrients in the green fodder will be conserved (Duniere et al., 2017; Gharechahi et al., 2017). Moreover, mowing the grassland at an optimal time for silage making can accelerate plant re-growth and improve forage biomass of grassland. In this case, mowing and ensiling the grasses from grassland could be a desirable feeding regime to alleviate the seasonal unbalanced feed supply problem on the QTP. It is well established that silage fermentation quality mainly depends on the microbial communities present and their succession during ensiling (McAllister et al., 2018; Ogunade et al., 2018). Various bacterial communities and successions were found in different pro- and after-ensiled forages (Parvin et al., 2010). Based on the previous studies, colonization of plant surfaces by bacteria depends on many factors including plant species, climate, vegetation period, geographical location, solar radiation intensity, and the type of fertilizer used (Pang et al., 2011; Mcgarvey et al., 2013). The harsh and unique environment even its variations in different areas on the QTP might lead to a different epiphytic microbial community of forages as compared to other regions worldwide. However, little information, to our best knowledge, is available on the epiphytic microbial community of forages from the grassland in different areas of the QTP, and its subsequent influences on silage fermentation and quality of native grasses. As a typical and important alpine forage grass, *Elymus nutans* is widely distributed in the QTP with high nutritional value and palatability for livestock (Chen et al., 2013; Xu et al., 2019).

Thus, the present study was conducted to investigate the epiphytic bacterial community, bacterial community dynamics, and natural fermentation of *E. nutans* silage prepared from four different regions of the QTP, which are the typical areas for raising the domestic animal of yaks on this plateau.

## Materials and Methods

### Study Sites and Silos Preparation

Grasses of *E. nutans* were harvested at heading stage from four different grasslands of Tianzhu County (TZ, Gansu province), Golog Prefecture (GL, Qinghai province), Damxung County (DX), and Nagqu Prefecture (NQ; Tibet Autonomous Region). These four places are typical areas for raising yaks on the QTP. The altitudes of these four grasslands used for harvesting the grass of *E. nutans* were 2965, 3763, 4228, and 4752 m, respectively. To make sure the grasses growing at different altitudes were harvested at the similar growth stage, the grasses from grasslands of GL, DX, and NQ were harvested from 22 to 25 August 2014; while the grasses from grassland of TZ were harvested on 6 September 2014. The grasses were harvested randomly at three sampling sites in each grassland. The grassland in each sampling area was selected based on good growth of *E. nutans*, and subsequently a size of 50 m × 100 m block was chosen as sampling site with vegetation coverage of above 90%. Three paired quadrats with frame sizes of 50 cm × 50 cm were selected for fresh forage sample collection. After harvesting, all the grasses were chopped as quickly as possible into 1–2 cm pieces using a paper cutter. The chopped grasses harvested at each sampling site were subsequently packed into polyethylene plastic bags with initial density of 0.52 g per cm^3^ (dimensions 270 mm × 300 mm; Embossed Food saver bag; Taizou Wenwu Soft-Packing Color-Printing Co., Ltd., Zhejiang, China) and vacuum-sealed tightly. Each polyethylene bag was packed with 200–230 g wilted grasses, with four replicates for each sampling site. The silos were then take back to the laboratory and stored at incubators (25 ± 0.2°C) for 14, 30, 60, and 90 days. Initial fresh forage samples were taken before ensiling.

### Chemical and Fermentation Profile Analyses

After opening the silos on each fermentation day, a portion of silage was immediately frozen (−20°C) in sealed plastic bags until further chemical analysis. A 20 g fresh weight (FW) from each sample was homogenized by using 180 mL of distilled water in a juice extractor (JYL-Y15, Joyoung Co., Ltd., Jinan, China). After squeezing for 30 s, the squeezed mash was filtered by using four layers of medical gauze (Sh&fe, Fu Zhou Medical Technology Co., Ltd., Fuzhou, China). The pH of filtrate was immediately measured by using a glass electrode pH meter (Hanna Instruments Italia Srl, Padua, Italy). The organic acids in the filtrate (lactic, acetic, propionic, and butyric acids) were then determined by using HPLC (KC-811 column, Shodex; Shimadzu: Japan; oven temperature 50°C; flow rate 1 mL min^–^^1^; SPD 210 nm) after acidulating a portion of the filtrate with H_2_SO_4_ (7.14 mol L^–^^1^) and filtering with a 0.22-μm dialyzer. About 250 g L^–^^1^ trichloroacetic acid was added to the remaining portion of the filtrate at a ratio of 1:4 and left overnight at 4°C for the precipitation of proteins. After centrifuging the protein precipitated solution at 18,000 × *g* for 15 min at 4°C, the supernatant fluid was used for analysis of ammonia nitrogen (NH_3_-N) and water-soluble carbohydrates (WSCs) concentrations as described by Ke et al. (2017).

The dry matter (DM) contents of the fresh and ensiled forages were determined by drying the samples in a forced-air oven at 65°C for 72 h. The dried samples were then ground by using a mill pulverizer (1 mm screen). Ground samples were analyzed for Kjeldahl N (AOAC, 1990) and the crude protein (CP) was calculated as Kjeldahl N × 6.25. The neutral detergent fiber (NDF) and acid detergent fiber (ADF) contents were analyzed by using an Ankom 2000 fiber analyzer (Ankom Technology, Fairport, NY, United States) according to the methods described by Van Soest et al. (1991) and Robertson and Van Soest (1981), respectively. When analyzing the fiber contents, the heat stable alpha amylase and sodium sulfite were added, and the values on NDF and ADF were expressed with residual ash.

### Enumeration of Microbial Populations

The methods described by Reich and Kung (2010) were used to enumerate LAB, yeasts, and molds in fresh and ensiled grass. Briefly, after opening the silos, a 10 g of the fresh forage or silage samples were immediately taken and homogenized in 100 mL of sterile Ringer’s solution (Oxide BR52) for 1 min, then serially diluted 10-fold. Enumeration of LAB was performed after spreading the 10-fold dilutions of water extracts in culture dish with MRS agar (Oxoid CM627, Oxoid, Basingstoke, United Kingdom) and incubation at 37°C for 48–72 h. Counts of yeasts and molds were enumerated on malt extract agar (Oxoid CM0059) that had been acidified with lactic acid (42.5 g L^–^^1^) after pour-plating serial 10-fold dilutions of water extracts and incubation at 32°C for 48–72 h. For enumeration of colonies in cultured plates, appropriate dilutions that yielded 30–300 colonies were counted.

### Microbial Composition SMRT Analyses

#### DNA Extraction

The PacBio single molecule, in conjunction with real-time sequencing technology (SMRT) was used to reveal the bacterial profile of target samples at the species level. Fresh and ensiled grasses fermented for 14, 30, 60, and 90 days were sampled for total bacteria DNA extraction. Fresh or ensiled grasses samples (15 g) from each treatment were diluted with 50 mL sterile saline water (0.85% NaCl) and shaken at a speed of 120 r min^–^^1^ for 30 min. Then 40 mL of each water extract was centrifuged at 10,000 r min^–^^1^ for 5 min to collect microorganisms. Total genomic DNA was extracted by using TIANamp Bacteria DNA Kit (DP302-02, Tiangen, China) following the manufacturer’s protocol. The concentrations of DNA samples were measured by a Thermo NanoDrop Spectrophotometer (ND-2000, United States). The quality of extracted DNA samples was evaluated by 1% agarose gel electrophoresis. All extracted DNA samples were stored at −20°C for further analysis.

#### PCR Amplification and SMRT Sequencing

The PCR amplification of the full-length 16S rRNA gene for SMRT sequencing was carried out by using the amplification program of: 94°C for 3 min, followed by 30 cycles of 94°C for 30 s, 55°C 30 s, and 72°C for 1 min, with final elongation of 72°C for 5 min. Reaction was performed in a 25 μL amplification mixtures with 12.5 μL Premix Taq^TM^ (TaKaRaTaq^TM^ Version 2.0, Takara Biotechnology), 0.5 μL of forward primer 27F (5’-GAGAGTTTGATCCTGGCTCAG-3’), 0.5 μL of reverse primer 1492R (5’-TACCTTGTTACGACTT-3’), 1 μL of DNA, and 10.5 μL of PCR-grade water. The quality control for PCR amplifications and sequences pre-processing were performed as described by Mosher et al. (2013). The 16S rRNA library was built with a Pacific Biosciences Template Prep Kit. Sequencing of the amplicons was performed on a PacBio Sequel instrument (Pacific Biosciences, Menlo Park, CA, United States). The SMRT sequencing and sequencing data analysis work were carried out by the company of Macrogen, Shengzhen sector, China, in year 2016.

### Sequences Analysis

Raw data were extracted and filtered with the Circular Consensus Sequencing (CCS) software of the SMRT Link (min full pass = 3, polish min predicted accuracy = 0.8, min length = 500) to obtain the Raw CCS Reads. The barcode reads of every sample were recognited with Lima^1^ to acquire Raw CCS, whereafter the CCS (accuracy above 99%) of each sample was tested and chimeric reads were removed with UCHIME^2^ to acquire optimized sequence. Subsequently, representative sequence was compared using the Mothur^3^ software with Silva (Release128^4^) database (classified at a bootstrap threshold of 0.9) to gain classified information (Quast et al., 2012). The Shannon–Wiener, Simpson’s diversity, Chao1, and rarefaction estimators were calculated to evaluate the alpha diversity. The sequence data reported in this study have been deposited in the NCBI Sequence Read Archive database (accession PRJNA623034^5^).

### Statistical Analyses

Data on silage fermentation and microbial populations of ensiled grasses at different fermentation times were subjected to the two-way analysis of variance using SAS software (SAS version 9.0, SAS Institute, Inc. Cary, NC, United States), and different fermentation times and sampling areas were used as two fixed factors. Microbial populations were estimated as colony-forming units (cfu) g^–^^1^ of either fresh grass or silage and were log transformed prior to statistical analysis. Data on chemical and microbial composition of fresh *E. nutans* before ensiling and on chemical composition of silages fermented for 90 days were subjected to one-way analysis of variance and polynomial contrast to examine effects of the unequally spaced elevation (by first generating the coefficients using PROC IML of SAS). Turkey’s multiple range test was used to separate means among treatments, and significance was declared at *P* < 0.05.

## Results

### Nutrient Composition and Microbial Population of the Fresh *E. nutans* Samples

The nutrient composition and microbial population of the fresh *E. nutans* samples from areas with different altitudes are shown in Table 1. The DM content of fresh *E. nutans* from four areas varied from 292.1 to 317 g kg^–^^1^. The WSC and CP contents linearly increased with an increase of altitude (*P* < 0.001). The contents of NDF and ADF in *E. nutans* decreased linearly with the increase of elevation (*P* < 0.001). The number of LAB in the samples from TZ was the lowest [5.48 log cfu g^–^^1^ of fresh matter (FM)] (*P* < 0.001), but abundant LAB were observed in the samples from GL and NQ (7.38 and 7.34 log cfu g^–^^1^ FM, respectively). The highest population of yeasts was found in samples from DX (6.66 log cfu g^–^^1^ FM), which was similar to that in samples from TZ (6.47 log cfu g^–^^1^ FM). The number of yeasts found in the samples from NQ was the lowest (5.00 log cfu g^–^^1^ of FM) (*P* < 0.001). On the contrary, the number of molds in the samples from NQ was the highest (3.78 log cfu g^–^^1^ FM) (*P* < 0.05).

**TABLE 1:** Characteristics of chemical and microbial composition of fresh *Elymus nutans* before ensiling.

	Site^2^					*P*-value	
Item^1^	TZ (2965 m)	GL (3763 m)	DX (4228 m)	NQ (4752 m)	SEM^3^	Linear	Quadratic
pH	6.29^b^	6.27^b^	6.25^b^	6.66^a^	0.06	<0.001	<0.001
DM (g/kg)	305.1^b^	292.1^c^	307.7^b^	317^a^	3.31	0.001	<0.001
WSC (g/kg DM)	63.9^c^	62.9^c^	86.9^b^	95.4^a^	5.38	<0.001	0.062
CP (g/kg DM)	75.1^c^	103.2^b^	106.8^b^	127.8^a^	6.93	<0.001	0.113
NDF (g/kg DM)	624.8^b^	652.6^a^	586.8^c^	572.2^d^	11.94	<0.001	<0.001
ADF (g/kg DM)	412.2^a^	351.2^b^	335.8^c^	333.9^c^	12.20	<0.001	<0.084
LAB (log cfu/g FM)	5.48^c^	7.34^a^	6.05^b^	7.38^a^	0.31	<0.001	0.001
Yeasts (log cfu/g FM)	6.47^a^	5.78^b^	6.66^a^	5.00^c^	0.23	<0.001	0.002
Molds (log cfu/g FM)	3.48^b^	3.60^b^	3.30^c^	3.78^a^	0.07	0.037	0.009

*^*a*−*c*^Means within a row without a common superscript letter differ. ^1^DM, dry matter; WSC, water-soluble carbohydrate; CP, crude protein; NDF, neutral detergent fiber (NDF assayed with a heat stable amylase and expressed inclusive of residual ash); ADF, acid detergent fiber; LAB, lactic acid bacteria. ^2^TZ, samples from grassland of Tianzhu County; GL, samples from grassland of Golog Prefecture; DX, samples from grassland of Damxung County; NQ, samples from grassland of Nagqu Prefecture. ^3^SEM, error of the means.*

### Fermentation Quality, Microbial Population, and Chemical Composition of *E. nutans* Silage

The pH values of the ensiled *E. nutans* samples from GL and NQ were rapidly reduced to approximately 4.4 after 14 days of ensiling. The pH values of the *E. nutans* silage samples from GL and NQ were lower than those from TZ and DX (Table 2; *P* < 0.05). After 60 days of ensiling, the concentration of lactic acid in the samples from NQ was lower than those in the *E. nutans* silage samples from the other three areas, but after 90 days, the concentration of lactic acid in the samples from NQ was higher than those from the other three areas (*P* < 0.05). Acetic acid was not detected in the *E. nutans* silage samples from TZ after 14 days of fermentation. After 60 days of fermentation, the concentration of acetic acid in the *E. nutans* silage samples from GL was higher than those in the *E. nutans* silage samples from the other three areas (*P* < 0.05). However, after 90 days of fermentation, the concentration of acetic acid in the *E. nutans* silage samples from NQ was the highest, whereas that from GL was the lowest.

**TABLE 2:** Fermentation characteristics of silage after 14, 30, 60 and 90 d^1^.

	Time		DM	LA	AA	LAB	Yeasts	Molds
Site^2^	(d)	pH	(g/kg DM)	(g/kg DM)	(g/kg DM)	(log cfu/g FM)	(log cfu/g FM)	(log cfu/g FM)
TZ	14	5.36	304.6	14.2	0.0	8.24	4.46	3.08
	30	4.34	292.8	12.4	4.3	8.51	4.00	3.10
	60	4.57	324.3	15.1	10.7	8.36	4.75	3.24
	90	4.29	282.8	50.0	13.1	8.32	4.96	3.10
GL	14	4.39	283.0	16.4	13.9	8.48	2.80	3.00
	30	4.28	273.6	21.0	9.6	8.39	2.48	0.00
	60	4.2	273.1	14.0	13.6	7.82	3.59	0.00
	90	4.11	276.1	55.3	10.2	7.58	3.14	0.00
DX	14	5.95	316.4	11.4	21.1	8.26	3.62	3.00
	30	4.88	333.4	17.1	5.2	8.81	4.51	3.07
	60	4.47	341.6	12.8	11.2	7.79	4.64	3.24
	90	4.28	336.9	40.2	15.3	7.43	4.65	3.00
NQ	14	4.41	331.8	15.6	13.7	5.74	3.06	0.00
	30	4.35	319.9	11.3	14.0	8.33	3.78	3.30
	60	4.18	316.8	9.8	10.9	7.85	4.61	3.30
	90	4.19	313.1	100.0	19.3	7.74	5.82	3.30
SEM^3^		0.03	0.814	0.634	0.654	0.208	0.114	0.066
*P*-value^4^	T	***	NS	***	**	NS	**	NS
	S	***	*	*	**	*	**	***
	T × S	***	NS	***	***	NS	NS	***

^1^DM, dry matter; FM, fresh matter; LA, lactic acid; AA, acetic acid; LAB, lactic acid bacteria. ^2^TZ, samples from grassland of Tianzhu County; GL, samples from grassland of Golog Prefecture; DX, samples from grassland of Damxung County; NQ, samples from grassland of Nagqu Prefecture. ^3^SEM, error of the means. ^4^S, site; T, ensiling time; S × T, interaction of S and T; NS, *P* > 0.05; *,0.01 < *P* < 0.05; **, 0.001 < *P* < 0.01; ***, *P* < 0.001.

There was no significant difference in the number of LAB in all samples (*P* > 0.05). The number of yeasts in the *E. nutans* silage samples from TZ and GL first decreased and then increased with the fermentation time, but the yeasts in the samples from DX and NQ showed an increasing trend. The number of yeasts in the samples from GL was the lowest (*P* < 0.05). After 90 days of fermentation, the number of yeasts in the samples from NQ was the highest (*P* > 0.05). Molds were found in the *E. nutans* silage samples from TZ, DX, and NQ during the fermentation process and their population did not change significantly (*P* > 0.05). No molds were detected in the samples from GL after 30 days of fermentation.

The nutrient composition of *E. nutans* silage samples from the four areas with different altitudes after 90 days of fermentation is shown in Table 3. The WSC content in the silage samples from TZ was the highest (8.23 g kg^–^^1^ of DM) and the WSC content linearly decreased with the increase of altitude (*P* < 0.05). The CP content in the samples increased linearly with the increase of altitude (*P* < 0.001); and decreases in NDF and ADF contents were observed in silage samples with the increase of altitude (linear and quadratic; *P* < 0.05). There was a linear decrease in silage NH_3_-N content with the increase of altitude from TZ to NQ (*P* < 0.001). The NH_3_-N contents in silage samples from TZ and GL were higher than those in the samples from DX and NQ (*P* < 0.05); however, there were no differences between samples from TZ and GL, and samples from DX and NQ as well (*P* > 0.05).

**TABLE 3:** Chemical composition of silages ensiled for 90 days^1^.

	WSC	CP	NH_3_-N	NDF	ADF
Site^2^	(g/kg DM)	(g/kg DM)	(g/kg TN)	(g/kg DM)	(g/kg DM)
TZ	8.23^a^	78.34^c^	114.7^a^	607.0^a^	363.2^a^
GL	4.26^bc^	106.7^b^	98.1^a^	629.9^a^	374.5^a^
DX	4.43^b^	113.4^b^	55.1^b^	609.6^a^	352.8^a^
NQ	3.14^c^	146.5^a^	49.6^b^	561.7^b^	318.5^b^
SEM^3^	0.396	4.79	7.62	6.16	5.36
*P*-value					
Linear	<0.001	<0.001	<0.001	0.001	<0.001
Quadratic	0.003	0.591	0.161	<0.001	0.009

*^*a*−*c*^Means within a column without a common superscript letter differ. ^1^DM, dry matter; WSC, water-soluble carbohydrate; CP, crude protein; NH_3_-N, ammonia nitrogen; TN, total nitrogen; NDF, neutral detergent fiber (NDF assayed with a heat stable amylase and expressed inclusive of residual ash); ADF, acid detergent fiber. ^2^TZ, samples from grassland of Tianzhu County; GL, samples from grassland of Golog Prefecture; DX, samples from grassland of Damxung County; NQ, samples from grassland of Nagqu Prefecture. ^3^SEM, error of the means.*

### Bacterial Community of Fresh and Ensiled *E. nutans* Samples

**FIGURE 1:**
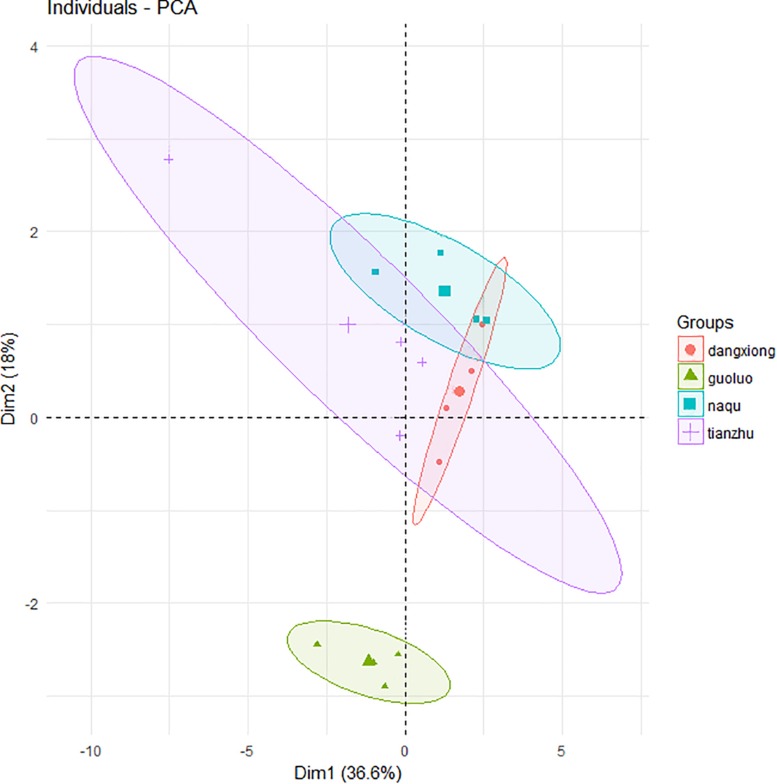
Principal component analysis (PCA) of bacteria community of fresh and ensiled forages. + Tianzhu County (TZ); ▲ Golog Prefecture (GL); ∙ Damxung County (DX); ■ Nagqu Prefecture (NQ). The big graphic symbols represent fresh grass, and the small graphic symbols represent ensiled grass.

A total of 27,821 SMRT sequencing reads were obtained from all silage samples based on SMRT sequencing of the full-length 16S rRNA gene of silage bacteria (Supplementary Table S1). The α-diversity (Chao1 index, Shannon index, Simpson index) and number of OTUs indicated a low bacterial biodiversity in the present *E. nutans* silage samples. The chao1 curves showed that the sequence depth was adequate for all samples (Supplementary Figure S1). Based on the principal component analysis of bacterial community results (Figure 1), there was one distinct cluster among the four different sampling sites. A clear separation by PCA between GL and the other three sites was observed, which represented 36.6% of variation among samples of fresh and ensiled forages.

**FIGURE 2:**
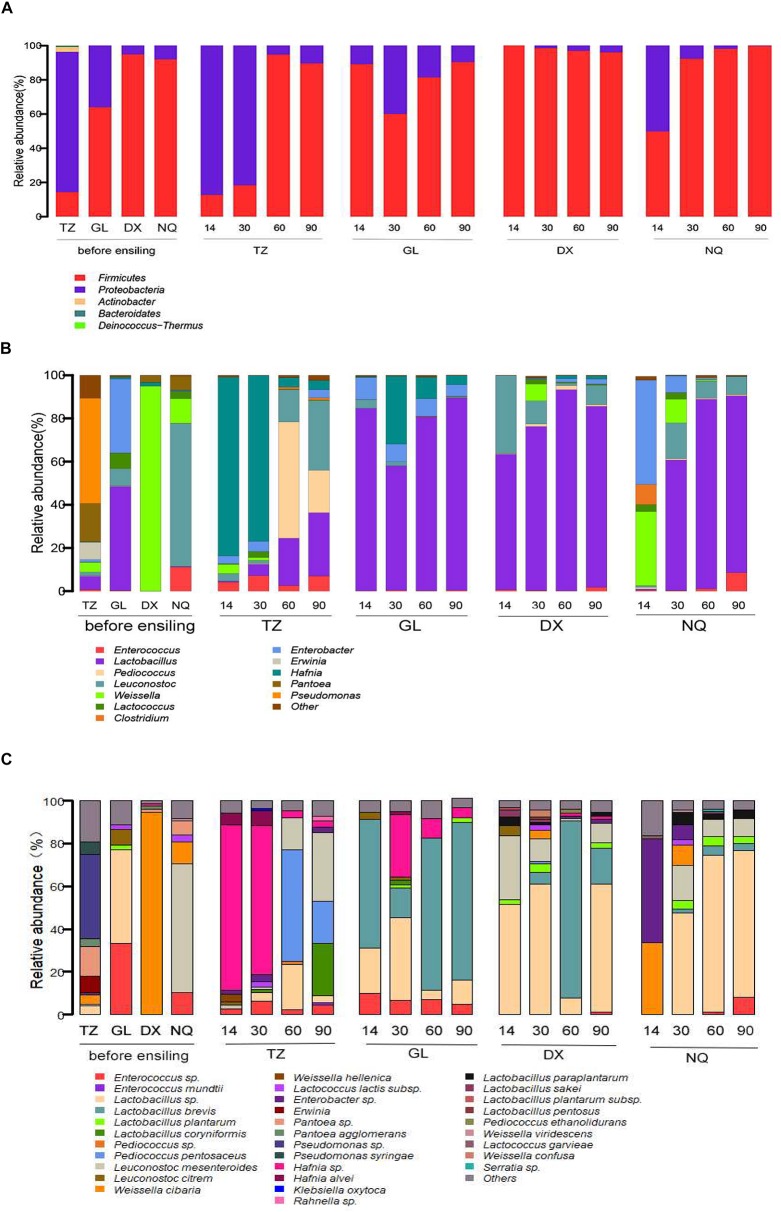
The epiphytic bacterial community of fresh *Elymus nutans* grass before ensiling and bacterial community of *E. nutans* silages fermented for 14, 30, 60, and 90 days. TZ, Tianzhu County; GL, Golog Prefecture; DX, Damxung County; NQ, Nagqu Prefecture. The bacterial communities were showed at the phylum level **(A)**, the genus level **(B)**, and the species level **(C)**.

The phylum-level changes in the bacterial composition of the *E. nutants* samples at different fermentation stages from the four sites with different altitudes (TZ, GL, DX, and NQ) are shown in Figure 2A. The main epiphytic microorganisms in the *E. nutans* samples were *Proteobacteria* and *Firmicutes*. In the *E. nutans* fresh samples from TZ, *Proteobacteria* was dominant, whereas in the fresh samples from GL, DX, and NQ, *Firmicutes* was dominant, especially in samples harvested from the high-altitude areas (DX and NQ) and its proportion was greater than 90%. During the silage fermentation process, the dominant bacteria in the 14 and 30 days samples from TZ was *Proteobacteria*, whereas the dominant bacteria changed to *Firmicutes* after 60 and 90 days of ensiling. The dominant bacteria in the samples from GL and DX were *Firmicutes* during the whole silage process. There was a large proportion of *Proteobacteria* in the 14 days samples from NQ but after 30 days of fermentation the *Firmicutes* became the dominant bacteria. At the end of the fermentation process, *Firmicutes* were dominant in all silage samples from the four areas.

The bacterial composition at the genus level is shown in Figure 2B. The dominant epiphytic bacteria associated with fresh *E. nutans* before ensiling were *Lactobacillus*, *Weissella*, *Erwinia*, *Pantoea*, *Pseudomonas*, *Leuconostoc*, *Lactococcus*, and *Enterobacter.* The microbial compositions of the fresh samples from the four different areas were different at the genus level. *Pseudomonas* and *Pantoea* were dominant in the fresh samples from TZ, and *Lactobacillus* and *Weissella* accounted for 6.12 and 4.45% of the population, respectively. *Weissella* was dominant in samples from DX, and *Leuconostoc* was dominant in samples from NQ. In the fresh samples from GL, *Lactobacillus* was the dominant bacteria and accounted for 48.22% of the population followed by *Enterobacter*.

After fermentation from 14 to 90 days, the dominant bacteria of *Weissella* in the fresh samples from DX was replaced by *Lactobacillus*. After 14 days of fermentation, the dominant bacteria of *Leuconostoc* in the fresh samples from NQ were replaced by species of *Weissella* and large portions of the undesirable bacteria of *Clostridium* and *Enterobacter* were also observed, whereas the *Leuconostoc* population was rapidly reduced to 0.79%. With the increase in fermentation time, *Lactobacillus* became the dominant bacteria and reached 81.91% of the population after 90 days of fermentation in the silage samples from NQ. Unfortunately, in the 14 and 30 days samples from TZ, *Hafnia* became the dominant bacteria, and until 60 and 90 days of fermentation, the bacteria composition was occupied by *Pediococcus*, *Lactobacillus*, and *Leuconostoc*. In samples from GL, *Lactobacillus* was the dominant bacteria during the whole fermentation process, whereas *Enterobacter* decreased from 34.26% in the fresh sample to 5.45% in the fermented sample of 90 days.

The bacterial composition at the species level is shown in Figure 2C. A total of 21 species of LAB were identified in the above six genera, the major species including *Enterococcus mundtii*, *Lactobacillus plantarum*, *Lactobacillus brevis*, *Pediococcus pentosaceus*, *Leuconostoc mesenteroides*, *Leuconostoc citreum*, *Weissella cibaria*, and *Lactococcus lactis*. The dominant species in the fresh grasses from TZ, GL, DX, and NQ were *Pseudomonas* (39.4%), *Lactobacillus* (43.7%), *W. cibaria* (94.5%), and *L. mesenteroides* (60%), respectively. In the samples from TZ, after 14 and 30 days of ensiling, *Hafnia* was the dominant bacteria, with proportions of 77.43 and 69.47%, respectively. Then the bacterial composition changed and mainly comprised by *P. pentosaceus*, *Lactobacillus* sp., *L. mesenteroides*, and *Lactobacillus coryniformis* after 60 days of ensiling. After the 14, 60, and 90 days of ensiling, *L. brevis* was the most abundant and accounted for 60.19, 71.02, and 73.76% of the population, respectively, in ensiled samples from GL. However, after 30 days of ensiling, *Lactobacillus* sp., *Hafnia*, and *L. brevis* were the dominant bacteria and accounted for 38.34, 29.29, and 14.18% of the population, respectively. Basically, *Lactobacillus* sp. and *L. brevis* were the dominant species in the samples from DX. The relative abundances of *Lactobacillus* sp. in 14, 30, and 90 days silage were 51.68, 60.87, and 59.61%, respectively; while, *L. brevis* was dominant and accounted for 82.75% of population in samples ensiled for 60 days. For the samples from NQ, *W. cibaria* and *Lactobacillus* sp. were dominant during fermentation. After 14 days of ensiling, *W. cibaria* increased to 33.83% of the population, whereas *Lactobacillus* sp. accounted for 47.51, 73.57, and 68.87% of the population in the 30, 60, and 90 days samples, respectively.

### Correlation Analysis Between Microbial Composition and Fermentation Quality of *E. nutans* Silage From Areas With Different Altitudes

**FIGURE 3:**
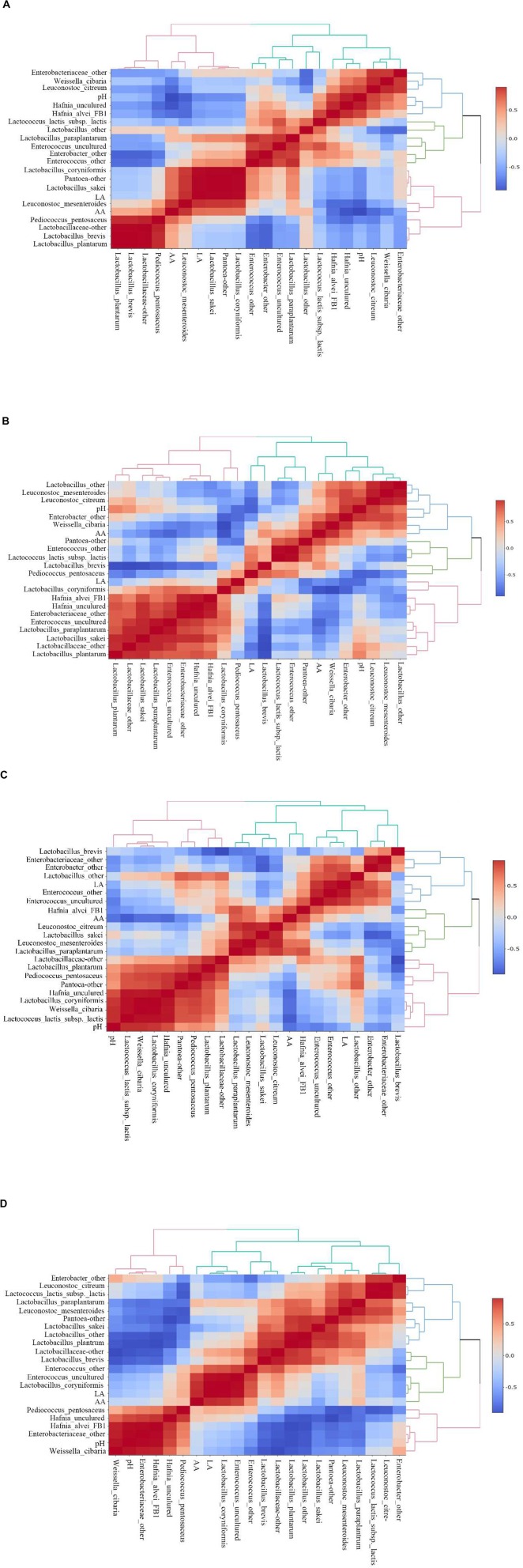
Spearman correlations and clustering analysis between fermentation parameters [pH, lactic acid (LA), acetic acid (AA)] and main bacteria species. The corresponding value of the middle heat map is the Spearman correlation coefficient *r*, which ranges between −1 and 1, *r* < 0 indicates a negative correlation (blue), *r* > 0 indicates a positive correlation (red). **(A)** Silage samples from Tianzhu County. **(B)** Silage samples from Golog Prefecture. **(C)** Silage samples from Damxung County. **(D)** Silage samples from Nagqu Prefecture.

Heatmaps of Spearman correlations between fermentation parameters and main bacteria species in *E. nutans* silage from different altitudes are shown in Figure 3. In the samples from TZ (Figure 3A), the acetic acid concentration was positively correlated with *L. mesenteroides*, *P. pentosaceus*, *Lactobacillus sakei*, *L. coryniformis*, and *Pantoea*-other, and was negatively correlated with *Hafnia alvei*, *Hafnia* uncultured, *L. citreum*, and *W. cibaria*. The concentration of lactic acid was positively correlated with *L. sakei*, *Pantoea*-other, *L. coryniformis*, and *L. Mesenteroides*. The pH value was positively correlated with *Hafnia* and negatively correlated with the AA content and *L. mesenteroides*. In the silage samples from GL (Figure 3B), the acetic acid concentration was positively correlated with *W. cibaria*, *Pantoea*-other, *Enterococcus*-other, *Enterobacter*-other, and *L. brevis*. The lactic acid concentration was positively correlated with *L. coryniformis* and *P. pentosaceus* despite of their low relatively abundances in the bacterial community. In the samples from DX (Figure 3C), the acetic acid concentration was positively correlated with *H. alvei*, *L. mesenteroides*, *L. citreum*, and *Lactobacillus paraplantarum*, and negatively correlated with *Hafnia* (uncultured), *L. lactis* subsp. *lactis*, and the pH. The concentration of lactic acid was positively correlated with *Enterococcus*, *Enterobacter*-other, and *Lactobacillus*-other. In the samples from NQ (Figure 3D), the concentration of acetic acid was positively correlated with *L. coryniformis* and *Enterococcus*. The lactic acid concentration was also positively correlated with *L. coryniformis* and *Enterococcus*.

## Disscusion

### Characteristics of Fresh *E. nutans* From Different Areas

The WSC is important energy for the growth of LAB, which decrease the pH (Moselhy et al., 2015). The increased WSC and CP in the fresh grass with increase of elevation may be due to the fact that the low temperature at high altitudes restrains plant respiration and thereby is favorable to accumulation of soluble carbohydrate, CP, and ether extract in cell protoplasm, which will decrease its freezing point and enhances its adaptive resistance to cold (Zhang and Ma, 1982). Previous study also reported that CP, crude fat, WSC, and other nutrients in forages increased with the increase of elevation (Guo et al., 2012). In addition, the low temperature could also prevent lignification of plant cell walls and decrease some structural carbohydrates such as cellulose, hemicellulose, and lignin (Zhao and Zhou, 1999; Xu et al., 2002).

The present result suggested that number of LAB in grass samples increased with the increase of altitude, and similar results were found in our previous study (Zhu et al., 2013). A variety of microorganisms are attached to plant surfaces, and plants can be spontaneously fermented after anaerobic silage. During fermentation, different silage materials harbor diverse microbes, both in terms of population and community composition (Khota et al., 2016). According to the previous reports, most epiphytic microorganisms degrade nutrients in plants, and among them, enterobacteria are dominant, followed by yeasts and molds; among epiphytic microorganisms, the proportion of beneficial microorganisms and undesirable microorganisms is approximately 1:10 (Lin et al., 1992). The number of epiphytic LAB on the plant is generally less than 5.0 log_10_ cfu g^–^^1^ FM (Pang et al., 2011). In this study, the number of epiphytic LAB in the *E. nutans* samples was over 5.0 log_10_ cfu g^–^^1^ FM. Because the grass is distributed in the QTP which is characterized by a harsh environment of low temperature, strong ultraviolet rays, and hypoxia, its epiphytic microorganisms may be adapted to the extreme environments.

### Fermentation Quality, Microbial Population, and Chemical Composition of *E. nutans* Silage

Ensiling is a complex bacterial fermentation process, which resulted in the accumulation of organic acid and decline of pH. In this study, the pH values of the ensiled *E. nutans* samples from GL and NQ were rapidly reduced. This is probably due to the higher amounts of LAB in grasses from GL and NQ as compared to that in the grasses from TZ and DX because green fodder is fermented by epiphytic LAB under anaerobic condition (Duniere et al., 2017). After 90 days fermentation, higher concentration of lactic acid was observed in the samples from NQ than those from the other three areas, but there was no significant difference in the number of LAB in all samples, indicating that the fermentation efficiency of LAB in the *E. nutans* silage samples from NQ may be greater in later fermentation stage. The acid production capacity can be further studied by screening and purifying the dominant LAB in the *E. nutans* samples from NQ. Acetic acid improves the aerobic stability of silage by inhibiting fungi (Kristensen et al., 2010). The concentration of acetic acid indicated various changes among different fermentation time in *E. nutans* silages from different areas in the present study. These changes might be due to the changes in bacterial composition or conservation of lactic acid to acetic acid during fermentation (Kung et al., 2018).

During the silage fermentation process, the abundance of LAB first increased and then decreased with fermentation time, which was in accordance with previous report (Ni et al., 2017). Molds were found in the *E. nutans* silage samples from TZ, DX, and NQ during the fermentation process, but no molds were detected in the samples from GL after 30 days of fermentation. This phenomenon might be due to that the LAB in the *E. nutans* samples from GL could produce strong antibacterial metabolites and inhibited the growth and reproduction of molds and other pathogenic bacteria (Amado et al., 2012).

As expected, the CP content increased and NDF, ADF, and WSC decreased with the increased altitude, which agrees well with previous findings (Guo et al., 2012). The NH_3_-N in silage is another predictor of silage fermentation quality, and it has been generally accepted that NH_3_-N in silage is decreased at lower pH (Jones et al., 1995). Higher NH_3_-N contents in silage samples from TZ and GL than those in the samples from DX and NQ were agreed well with the present results on silage pH.

### Microbial Composition of *E. nutans* Before and After Ensiling

Ensiling without application of inoculants or starter cultures is a spontaneous fermentative process in which the fermentation mainly depends on the epiphytic microbial composition and the natural occurrence of epiphytic LAB (Guo et al., 2018). The bacterial community and its dynamics in the spontaneous fermentative process of ensiled *E. nutans* made in the QTP have rarely been reported. Metagenomic approaches, such as terminal restriction fragment length polymorphism, denaturing gradient gel electrophoresis, and 454 high-throughput sequencing, have been widely used to track the bacterial communities in ensiled forages in the past decade (Mcgarvey et al., 2013). However, these techniques either only reflect a few of the most abundant operational taxonomic units (OTUs) present or restrict to genus-level identification because only a partial sequence of the 16S rRNA gene is evaluated. As it was reported that by using the PacBio SMRT method, the bacterial composition and its dynamics in ensiling process can be precisely revealed at the species level because it can generate long sequence reads, which provides valuable biological information regarding the complete bacterial community in the ensiling system (Guo et al., 2018). Therefore, the PacBio SMRT method was used to analyze the bacterial changes in the fermentation process of ensiled *E. nutans* at the phylum, genus, and species levels in the present study.

The fresh *E. nutans* was dominated by *Firmicutes* especially in the high-altitude areas (DX and NQ) with abundance over 90%. Therefore, it could be hypothesized that *Firmicutes* is more resistant to extreme environments and can adapt to the unique high-altitude habitat of the QTP. In addition, *Firmicutes* was also dominated the whole fermentation process in the samples from GL and DX, and similar result was reported in barely silage (Liu et al., 2019). Previous study also reported that the abundance of *Firmicutes* increased but the abundance of *Proteobacteria* decreased in ensiled forages during ensiling (Mcgarvey et al., 2013).

At genus level, the dominant epiphytic bacteria associated with fresh *E. nutans* were *Lactobacillus*, *Weissella*, *Erwinia*, *Pantoea*, *Pseudomonas*, *Leuconostoc*, *Lactococcus*, and *Enterobacter*, which are different from the dominant epiphytic bacteria of *Lactobacillus*, *Acetobacter*, *Weissella*, *Pseudomonas*, *Acinetobacter*, and *Burkholderia* reported in fresh corn cultivated in low altitude region of Southwestern China (Guan et al., 2018), and the dominant epiphytic bacteria of *Enterobacter*, *Pseudomonas*, and *Pantoea* reported in soybean stalk before ensiling (Ni et al., 2017, 2018), and also the dominant epiphytic bacteria of *Pantoea* (34.7%), *Weissella* (28.4%), *Pseudomonas* (10.4%), *Exiguobacterium* (7.8%), and *Paenibacillus* (3.4%) in fresh wheat before ensiling from the temperate oceanic climate of Germany (Keshri et al., 2019). It was suggested that altitude and climate may affect the distribution of LAB (Leong et al., 2014). In the present study, the bacterial composition of the samples from TZ was dominated by *Pseudomonas*, *Pantoea*, *Erwinia*, *Lactobacillus*, and *Weissella*, which was more complex than that of the samples from DX and NQ. Although no study, to the best of our knowledge, has been conducted to investigate effect of altitude on bacterial composition in fresh forage, it has been reported that *Weissella* and *Leuconostoc* are commonly found LAB species in forage crops and silage (Cai et al., 1998). In accordance with the previous report (Pahlow et al., 2003), the LAB in *E. nutans* silage included six genera: *Enterococcus*, *Lactobacillus*, *Pediococcus*, *Leuconostoc*, *Weiss*, and *Lactococcus*; however, these genera had a various distribution in *E. nutans* silages made from the four different areas in the present study. After 14 days of fermentation, the dominant bacteria were replaced by homofermentative species of *Lactobacillus*, which promoted silage fermentation. This result is consistent with the acid production in the silage samples from DX and NQ, and previous studies also indicated that *Lactobacillus* was the dominant bacteria in ensiled forages (Guan et al., 2018; Liu et al., 2019; Yan et al., 2019). However, some undesirable bacteria like *Hafnia* and *Enterobacter* dominated the ensiled *E. nutans* made in TZ from 14 to 30 days of fermentation. The result indicated that the fermentation of samples from TZ was slow, and those undesirable bacteria for silage fermentation were competitive for a relative long time during ensiling. A possible reason is that the epiphytic bacteria in the fresh forage was mostly comprised by undesirable microbes, such as *Pseudomonas*, *Hafnia*, and *Erwinia*, which inhibited the fermentation by LAB until 30 days. In the ensiled *E. nutans* samples from GL and DX, the *Lactobacillus* occupied the bacterial community from 14 to 90 days of ensiling, which was probably related to the initially high proportions of *Lactobacillus* or *Weissella* in the epiphytic bacteria of fresh *E. nutans* harvested from GL and DX, respectively. The *Leuconostoc*, *Enterococcus*, and *Weissella* dominated epiphytic bacterial community in fresh *E. nutans* harvested from NQ probably resulted in the undesirable bacterial community mainly comprised by *Enterobacter*, *Pseudomonas*, and *Weissella* in the 14 days silage, but the bacterial community was dominated by *Lactobacillus* after 30 days of ensiling.

In the present study, the significant differences at the species level in the epiphytic microorganisms of the *E. nutans* fresh samples from the four areas were observed. Therefore, the LAB succession at different fermentation stages of *E. nutans* was also significantly different. The dominant bacteria in the fresh grasses from TZ, GL, DX, and NQ were *Pseudomonas*, *Lactobacillu*, *W. cibaria*, and *L. mesenteroides*, respectively, which was also different from previous studies on fresh whole crop corn, grass, and legume forages (Ni et al., 2017; Guan et al., 2018; Liu et al., 2019; Xu et al., 2019; Yan et al., 2019). Moreover, the LAB composition in *E. nutans* silage is totally different that in whole crop corn silage (Xu et al., 2019). Results from the present study showed that the bacteria were mainly comprised by *P. pentosaceus*, *Lactobacillus* sp., *L. mesenteroides*, and *L. coryniformis* after 60 days of fermentation in the silage samples from TZ, while *L. brevis* was the dominant species in the silage samples from DX after the 14, 60, and 90 days fermentation. Generally, *Lactobacillus* sp. and *L. brevis* were the dominant species in the samples from DX, whereas in the samples from NQ, *W. cibaria* and *Lactobacillus* sp. were dominant during fermentation. Our previous study indicated that *L. plantarum* was the most abundant LAB species in ensiled alfalfa during the spontaneous fermentation from 30 to 90 days (Guo et al., 2018). The bacteria in fresh alfalfa before ensiling included *Enterobacter*, *Erwinia*, and *Pantoea*, whereas *Lactobacillus*, *Pediococcus*, and *Lactococcus* were the primary bacteria after ensiling (Mcgarvey et al., 2013). Guan et al. (2018) showed that *Weissella* was the dominant bacteria in whole plant corn silage, and *Lactobacillus* and *Acetobacter* were the dominant LAB after fermentation. As it was reported that the composition of bacteria on plant surfaces depends on many factors, including plant species, climate, geographic location, and types of fertilizer used (Mcgarvey et al., 2013).

### Correlations Between Main Bacteria Species and Fermentation Quality

The correlations between acetic or lactic acids and bacteria in silage samples form TZ indicated that LAB species of *L. mesenteroides*, *P. pentosaceus*, *L. sakei*, and *L. coryniformis* probably played an important role in the fermentation of ensiled *E. nutans* harvested from TZ. In addition, because *P. pentosaceus* is a homofermenter (Schmidt and Kung, 2010), the species of *L. mesenteroides* was proposed as a major contributor for producing acetic acid during fermentation. Previous study reported that *acetobacter* was the major contributor for producing acetic acid in natural fermented whole crop corn silage made in Southwest China (Guan et al., 2018). High relative abundances of *L. brevis* and *Lactobacillus* sp. were observed in samples from GL. In this case, these two species should be the major contributors for production of lactic acid and acetic acid in the silage samples from GL because *L. brevis* is known as hetero-fermentative strain (Daniel et al., 2015). Therefore, in *E. nutans* silages made from GL, the LAB species of *L. brevis*, *Lactobacillus* sp., *L. coryniformis*, and *W. cibaria* were the major contributors for producing acetic acid and lactic acid, respectively. According to the correlations between fermentation quality and microbes in silage samples form TZ and GL, *L. mesenteroides*, *P. pentosaceus*, *L. sakei*, *L. coryniformis*, *L. brevis*, and *W. cibaria* could be candidate LAB species for developing inoculants to improve silage quality in TZ and GL of the Tibetan plateau.

The correlations between acetic or lactic acids and microbes in silage samples form DX suggested that *Lactobacillus* sp., *Enterococcus*, *L. mesenteroides*, and *L. brevis* (high relative abundance in 60 days silage) were the major contributors for producing acetic acid and lactic acid, respectively. Considering the high relative abundances of *Lactobacillus* sp., *W. cibaria*, *L. brevis*, *L. plantarum*, and *L. mesenteroides* in ensiled forage harvested from NQ, the major contributors for producing acetic acid and lactic acid in silages from NQ could be *Lactobacillus* sp., *W. cibaria*, *L. brevis*, *L. plantarum*, *L. mesenteroides*, *L. coryniformis*, and *Enterococcus*. However, the functional features of a small number of species can also have a large impact on community structure and ecosystem functioning (Shabat et al., 2016). Therefore, the LAB species, such as *W. cibaria*, *L. brevis*, *L. mesenteroides*, *L. plantarum*, and *L. coryniformis* could be candidate LAB species for developing inoculants to improve silage quality in DX and NQ of the Tibetan plateau.

The results of correlation analysis between bacterial composition and fermentation quality of *E. nutans* silages made from the four different areas in the QTP further indicated that the LAB species responsible for silage fermentation in different areas were different, which was mainly due to the different epiphytic bacterial compositions in fresh *E. nutans* before ensiling. However, the differences in fermentation and bacterial composition can not only be the geographical origin, factors like altitude, climate, solar radiation intensity, and period of vegetation may also affect the distribution of epiphytic bacteria of fresh forages (Mcgarvey et al., 2013; Leong et al., 2014) and consequently influence the distribution LAB after ensiling. Additionally, the present results also provided important information for screening suitable LAB species to make high quality *E. nutans* silage in the four different areas. Inoculants of LAB are often and widely used to improve silage fermentation quality. On the QTP, however, the average diurnal temperature at the stage of forage harvest is about 10–15°C. The low temperature is considered as a main limitation factor for commercial inoculants to work effectively on this plateau because the commercially available LAB strains are generally isolated from temperate environment and adapted to at a moderate high temperature (Zahar et al., 2002). Therefore, screening the indigenous LAB strains from the plateau to improve silage fermentation quality on the QTP is highly recommended.

## Conclusion

The fermentation quality of *E. nutans* from different areas (TZ, GL, DX, and NQ) and the changes in bacterial composition during fermentation were varied based on the composition of epiphytic microbes of *E. nutans*. The dominant species in the fresh grasses from TZ, GL, DX, and NQ were *Pseudomonas* (39.4%), *Lactobacillus* (43.7%), *W. cibaria* (94.5%) and *L. mesenteroides* (60%), respectively. The LAB involved in fermentation of *E. nutans* from low altitude areas included *P. Pentosaceus*, *Lactobacillus* sp., *L. mesenteroides*, and *L. coryniformis*, whereas major LAB species involved in the fermentation of *E. nutans* silage from high altitudes included *L. mesenteroides*, *L. brevis*, and *Lactobacillus* sp. Correlation analysis between bacterial composition and fermentation quality of *E. nutans* silages made from the four different areas in the QTP indicated that the LAB species responsible for silage fermentation in different areas were different, which was mainly due to the different epiphytic bacterial compositions in fresh *E. nutans* before ensiling. These results provide important information on revealing the bacterial communities and fermentation qualities of ensiled *E. nutans* sampled from four different areas, and on future screening of LAB isolates for making high quality silage to alleviate feed shortage of the traditional year-round grazing system on the QTP.

## Data Availability Statement

The datasets analyzed in this manuscript are not publicly available. Requests to access the datasets should be directed to guoxsh07@lzu.edu.cn.

## Author Contributions

ZD, JB, and XG designed the study and wrote the manuscript. ZD, DX, JB, FL, and YZ performed the experiments. ZD, DX, and XG analyzed the data. All authors reviewed the manuscript.

## Conflict of Interest

The authors declare that the research was conducted in the absence of any commercial or financial relationships that could be construed as a potential conflict of interest.

## Footnotes

1 https://github.com/PacificBiosciences/barcoding

2 http://drive5.com/usearch/manual/uchime_algo.html

3 https://mothur.org/wiki/Classify.seqs

4 http://www.arb-silva.de

5 http://www.ncbi.nlm.nih.gov/sra/
