# Supplementary material for: Microbial Community Dynamics and Natural Fermentation Profiles of Ensiled Alpine Grass Elymus nutans Prepared From Different Regions of the Qinghai-Tibetan Plateau
Source: Front Microbiol. 2020 May 12;11:855. doi: 10.3389/fmicb.2020.00855 (PMC7235320; doi:10.3389/fmicb.2020.00855)
Supplement: Supplementary file 1 [file Data_Sheet_1.docx]

***Supplementary Materials***

**Microbial community dynamics and natural fermentation profiles of ensiled alpine grass *Elymus nutans* prepared from different regions of the Qinghai-Tibetan plateau**

Zitong Ding^1,2#^, Jie Bai^2,3#^, Dongmei Xu^1,2^, Fuhou Li^2,3^, Yixin Zhang^1,2^, Xusheng Guo^1,2*^

^1^State Key Laboratory of Grassland Agro-ecosystems, School of Life Sciences, Lanzhou University, Lanzhou 730000, PR China.

^2^Probiotics and Biological Feed Research Centre, Lanzhou University, Lanzhou 730000, PR China.

^3^State Key Laboratory of Grassland Agro-ecosystems, College of Pastoral Agriculture Science and Technology, Lanzhou University, Lanzhou 730020, PR China.

^#^ These authors contributed equally to this work.

*Corresponding authors: Dr. Xusheng Guo; School of Life Sciences, Lanzhou University, No. 222 Tianshui South Road, Lanzhou 730000, PR China.

Tel.: +86 931 8915650;

Fax: +86 931 8915650.

E-mail addresses: [guoxsh07@lzu.edu.cn](mailto:guoxsh07@lzu.edu.cn) (X.S. Guo).

**TABLE S1 Sequence and bacterial diversity of fresh forage and experimental treatment groups.**^1^TZ, samples from grassland of Tianzhu County; GL, samples from grassland of Golog Prefecture; DX, samples from grassland of Damxung County; NQ, samples from grassland of Nagqu Prefecture. ^2^ represent different fermentation times; 0 means fresh forage; ^3^Goods coverage: coverage is calculated as C=1-(*s*/*n*), where *s* is the number of unique OTUs and *n* is the number of individuals in the sample. This index gives a relative measure of how well the sample represents the larger environment.

| Site^1^ | Time^2^ (d) | Number of reads | Average read length | Number of OTUs | Chao 1 index | Shannon index | Simpson index | Goods  coverage^3^ |
| --- | --- | --- | --- | --- | --- | --- | --- | --- |
| TZ | 0  14 | 4057 2344 | 1506  1499 | 149  98 | 154.2  143.1 | 4.978  3.578 | 0.91  0.78 | 0.92  0.94 |
|  | 30 | 1485 | 1496 | 61 | 96.7 | 3.833 | 0.82 | 0.95 |
|  | 60 | 1246 | 1520 | 41 | 75.5 | 2.856 | 0.74 | 0.94 |
|  | 90 | 1151 | 1517 | 37 | 59.7 | 3.199 | 0.80 | 0.92 |
|  |  |  |  |  |  |  |  |  |
| GL | 0  14 | 1463  1813 | 1511  1523 | 61  65 | 109.4  175 | 2.88  2.61 | 0.73  0.63 | 0.94  0.93 |
|  | 30 | 1916 | 1514 | 76 | 145.2 | 3.62 | 0.83 | 0.91 |
|  | 60 | 1840 | 1517 | 69 | 155.8 | 2.19 | 0.49 | 0.93 |
|  | 90 | 1965 | 1520 | 73 | 171.1 | 2.13 | 0.47 | 0.94 |
|  | 0 | 2324 | 1528 | 24 | 29.1 | 0.54 | 0.11 | 0.99 |
| DX | 14 | 1250 | 1517 | 44 | 77.3 | 2.55 | 0.67 | 0.95 |
|  | 30 | 1312 | 1517 | 47 | 70 | 2.99 | 0.68 | 0.93 |
|  | 60 | 1949 | 1522 | 56 | 94.1 | 1.81 | 0.37 | 0.94 |
|  | 90 | 1746 | 1523 | 63 | 141.2 | 2.95 | 0.70 | 0.92 |
|  | 0 | 812 | 1505 | 27 | 53.3 | 2.38 | 0.63 | 0.95 |
| NQ | 14 | 2564 | 1504 | 47 | 48.1 | 2.52 | 0.69 | 0.99 |
|  | 30 | 1298 | 1520 | 47 | 122.6 | 3.34 | 0.79 | 0.92 |
|  | 60 | 1687 | 1520 | 57 | 109.8 | 2.50 | 0.55 | 0.94 |
|  | 90 | 1730 | 1523 | 56 | 94.2 | 2.53 | 0.59 | 0.95 |

**
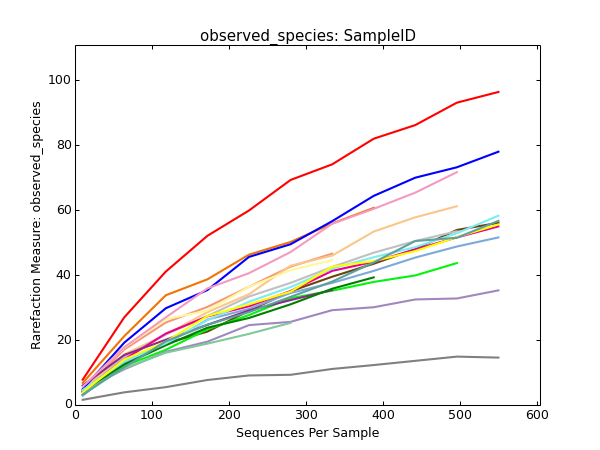
** **
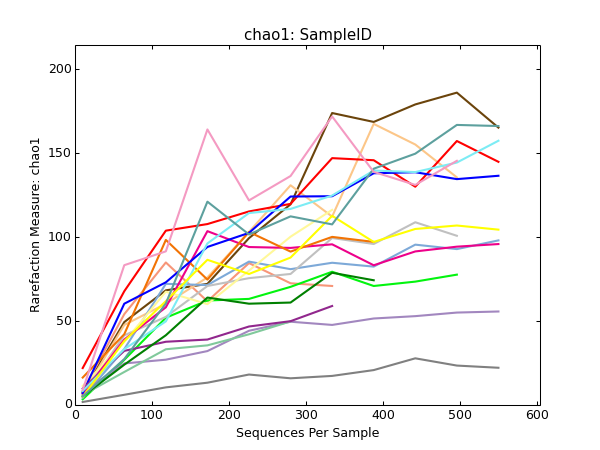
**

**Figure S1.** The rarefaction curves and chao 1 diversity index curves of the 16S rRNA gene reads derived from the observed species.
